# Multiple objectives optimization of injection-moulding process for dashboard using soft computing and particle swarm optimization

**DOI:** 10.1038/s41598-024-62618-7

**Published:** 2024-10-10

**Authors:** Mehdi Moayyedian, Mohammad Reza Chalak Qazani, Parisa Jourabchi Amirkhizi, Houshyar Asadi, Mohsen Hedayati-Dezfooli

**Affiliations:** 1 College of Engineering and Technology, American University of the Middle East, Egaila, 54200 Kuwait; 2 Faculty of Computing and Information Technology (FoCIT), Sohar University, Sohar, 311 Oman; 3 Design Faculty, Tabriz Islamic Art University, Tabriz, Iran; 4 Institute for Intelligent Systems Research and Innovation (IISRI), Deakin University, Waurn Ponds, VIC 3216 Australia; 5 Department of Mechanical Engineering, College of Engineering and Technology, University of Doha for Science and Technology, Arab League St, 24449 Doha, Qatar

**Keywords:** Injection moulding, Warpage/shrinkage/sink mark, Soft computing, Multiple objectives particle swarm optimisation, Pareto front, Engineering, Mechanical engineering

## Abstract

This research focuses on utilizing injection moulding to assess defects in plastic products, including sink marks, shrinkage, and warpages. Process parameters, such as pure cooling time, mould temperature, melt temperature, and pressure holding time, are carefully selected for investigation. A full factorial design of experiments is employed to identify optimal settings. These parameters significantly affect the physical and mechanical properties of the final product. Soft computing methods, such as finite element (FE), help mitigate behaviour by considering different input parameters. A CAD model of a dashboard component integrates into an FE simulation to quantify shrinkage, warpage, and sink marks. Four chosen parameters of the injection moulding machine undergo comprehensive experimental design. Decision tree, multilayer perceptron, long short-term memory, and gated recurrent units models are explored for injection moulding process modelling. The best model estimates defects. Multiple objectives particle swarm optimisation extracts optimal process parameters. The proposed method is implemented in MATLAB, providing 18 optimal solutions based on the extracted Pareto-Front.

## Introduction

Plastic injection moulding process involves several stages. Initially, the polymer material and additives are fed into the heating system of the injection moulding machine. Subsequently, the heated polymer is injected into the mould cavity during the filling phase. In the packing stage, additional polymer melt is applied at elevated pressure to offset shrinkage. The mould is then subjected to cooling until the part solidifies. Ultimately, the mould is opened, and the plastic part is ejected from the cavity using ejector pins, marking the completion of one cycle, after which the process is repeated^1–3^.

The quality of injected products is inherently uncertain. Operators typically develop a wealth of experience to determine the optimal combination of process parameters. In injection processing, a strong correlation exists between process parameters and the quality of the injected part. Improper configuration of these parameters can lead to various product defects, including sink marks, shrinkage, and warpages^4^. Warpage and shrinkage are common issues in injection moulding. Shrinkage refers to reducing the size of a plastic part as it cools. At the same time, a warpage is the deformation of a part due to uneven cooling^5^. A study focuses on the impact of process parameters on warpage and shrinkage in thin-wall technology. The study validates the procedure using a real case of moulding a cell phone shell with PC/ABS material. The results demonstrate accurate predictions of shrinkage and warpage effects using quadratic models.

By applying the optimal procedure, significant reductions in shrinkage (37.8%) and warpage (53.9%) are achieved for PC/ABS cell phone shells^6^. Sink marks in injection moulding refer to depressions or indentations that appear on the surface of a moulded part due to uneven shrinkage during the cooling phase. Sink marks commonly occur behind the ribs^7^. A theoretical model incorporating rib design and processing parameters was developed to analyse sink marks. Design of experiments^8^, FE flow analysis^9^, and GA^10^ were used. Sink mark depth depends on design variables and technological parameters. Four key variables (rib thickness, mould temperature, melt temperature, and coolant temperature) were optimized using the prediction model and GA to minimize sink depth^11^.

Pandelidis and Kao^12^ Developed KBS for diagnosing multiple defects in injection moulding using FIS, with an efficient algorithm for selecting the best cover of causes and providing remedies based on material properties. Liu et al.^13^ introduced a SNCCDBAGG-based neural network ensemble approach for quality prediction in the injection moulding process, combining bagging, negative correlation learning, and a selection-based strategy to improve generalization ability and achieve enhanced performance compared to single neural network and negative correlation learning predictors. Zhao^14^ developed an automatic QSSPP algorithm for phase-based regression modelling and quality prediction in batch manufacturing processes, particularly illustrated through injection moulding, thereby addressing the challenge of partitioning phases and capturing time-varying quality prediction relationships. Moayyedian et al.^15^ introduced a modified edge gate in injection moulding, aimed at reducing internal and external defects, improving de-gating analysis, and achieving a significant reduction in scrap rates compared to the current edge gate. The used ANOVA to assess the geometric parameters and process effect on scrap rate. Jha et al.^16^ developed a comprehensive prognostic solution for industrial proton exchange membrane fuel cells, integrating bond graph theory and particle filters for accurate remaining useful life prediction. Tsai et al.^17^ developed a hybrid model combining GA and MLP for establishing an inverse model of injection moulding, successfully identifying process parameters for achieving reliable lens form accuracy with a significant improvement rate. Abbasalizadeh et al.^18^ investigated the influence of injection moulding parameters on the shrinkage of different polymers, highlighting the significant influence of material crystallinity and flow direction. Optimum conditions for minimizing shrinkage were determined using the Taguchi approach. Khosravani et al.^19^ reviewed the implementation of case-based reasoning as a soft computing method in the injection moulding process. Abdul et al.^20^ developed an MLP model combined with the Taguchi approach to predict and minimize part shrinkage in injection moulding, improving quality and facilitating the moulding setup process. Song et al.^21^ developed a hybrid model combining GA, MLP, and SVR to optimise design parameters and accurately predict warpage and volume shrinkage in injection moulding. Gao et al.^22^ proposed soft computing methods (MLP, SVR, and kernel ridge) for conformal cooling channels in injection moulding, resulting in reduced temperature variance and improved cooling quality compared to conventional designs. Li et al.^23^ proposed prediction models based on Taguchi and multiple linear regression techniques to minimize dimensional deviation in injection moulding. Jung et al.^24^ evaluated the effectiveness of different soft computing techniques in predicting the quality of injection moulding. Uğuroğlu^25^ introduced a real-time application for plastic injection moulding machines, employing soft computing methods such as k-nearest neighbour, random forest, logistic regression, and MLP. Zhang et al.^26^ systematically analysed and optimised injection moulding parameters for replicating microlens arrays for light-field applications using Gaussian regression filter. Li et al.^27^ introduced an off-policy reinforcement learning approach for fault-tolerant control in industrial processes, without requiring knowledge of system dynamics. Wang et al.^28^ used a reinforcement learning approach to optimise a fault-tolerant tracking control for industrial processes, improving performance and expanding fault tolerance.

Recently, there has been a notable concentration on enhancing the efficiency of process parameters in injection moulding. Lockner et al.^29^ introduced a transfer learning to improve injection moulding process modelling with limited data, resulting in higher model quality. Párizs et al.^30^ conducted a comparison of various soft computing methods to forecast the quality of multi-cavity injection moulding. Their results showed that the DT model achieved the highest accuracy, exceeding 90%. Ke and Huang^31^ introduced an optimised MLP model with a Sigmoid activation function and a learning rate 0.1, achieving an accuracy of 95.8%. Moayyedian et al.^32^ developed a computationally efficient model using genetic programming to optimise injection moulding parameters. Their approach demonstrated significantly lower Mean Squared Error (MSE) compared to previous methods such as SVR, DT, and MLP. Gim et al.^33^ employed transfer learning techniques to optimise process parameters for achieving high surface quality in injection moulding. By training a multi-task MLP model on data from the original production site and transferring it to a new site, they improved surface gloss prediction, reduced dataset size. They enabled the efficient production of high-quality moulded parts. Wu et al.^34^ introduced a generative soft computing-based multi-objective optimisation model for injection moulding. This model predicted part qualification and optimised processing variables to minimize weight difference and energy consumption. It enhanced the quality assurance and energy efficiency of plastic manufacturing.

In general, the investigated soft computing methods are KBS^12,19^, ANOVA^15^, linear regression^23–26^, MLP^17,20–22,25,31,32^, SVR^21,22,24,32^, random forest^24,25,34^, DT^24,30,32^, k-nearest neighbour^25,30^, genetic programming^32,34^, and deep learning^27–29,33^. In addition, mathematical^32^ and meta-heuristic^16,17,21,34^ optimisations are used to extract the optimal injection parameters for producing different parts. However, there is a lack of comprehensive soft computing investigation in modelling the injection moulding process of the complicated parts, such as a dashboard considering different injection moulding process parameters. The main novelty of the current research can be categorised into two different points:

Developing comprehensive soft computing methods, including DT, MLP, LSTM, and GRU, for estimating injection moulding defects (shrinkage, warpage, and sink marks) based on process parameters (melt temperature, mould temperature, pressure holding time, and pure cooling time).

The implementation of multiple objectives particle swarm optimisation (MOPSO) with consideration of three objective functions, including shrinkage, warpage, and sink marks.

**Figure 1:**
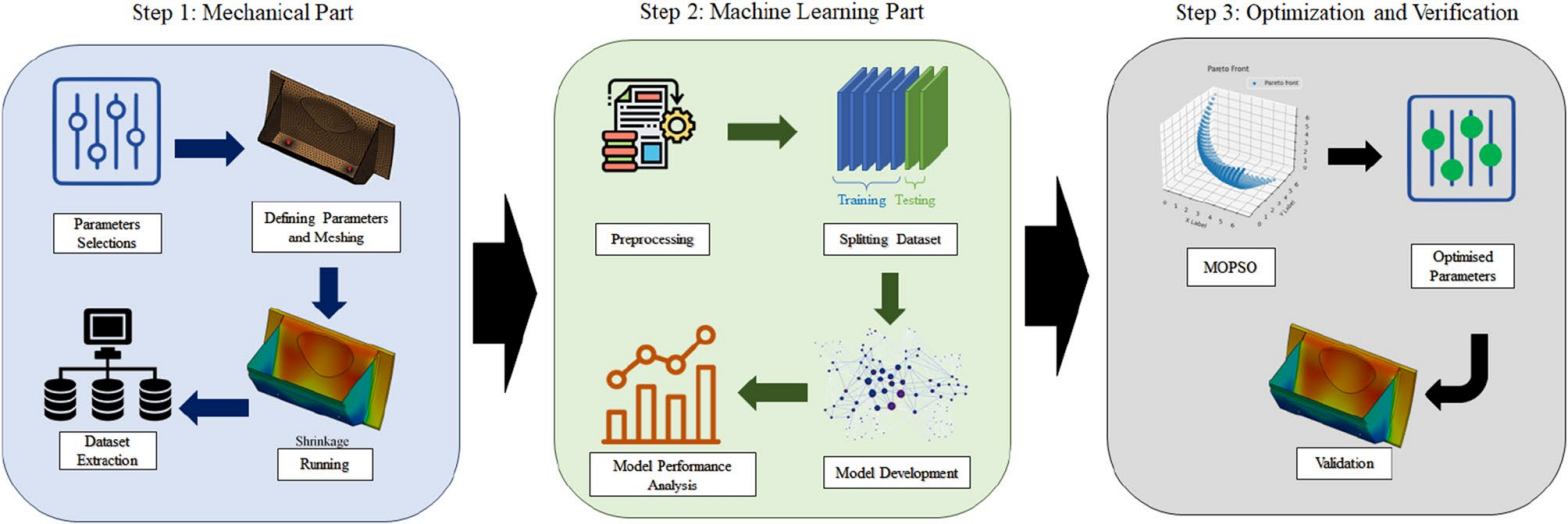
The overview of the proposed method in this study for extracting the optimal injection moulding process parameter for dashboard production.

The datasets are generated via the FE simulation environment, SOLIDWORKS (SW) Plastics. The input parameters of the simulations are defined as melt temperature, mould temperature, pressure holding time, and pure cooling time. While the results are recorded via the shrinkage, warpage, and sink marks defects of the injection moulding. The recorded dataset is used for training/validation/testing purposes of the investigated soft computing techniques (DT, MLP, LSTM, and GRU). Then, based on higher accuracy, three models are developed for calculating injection moulding defects. In the last step, these three extracted soft computing methods are employed inside the MOPSO to extract the 3-dimensional Pareto front of the optimal solutions. The input process parameters of the optimal 3-dimensional Pareto front are recommended to reach the higher efficiency of the process in the last part of the article after validation with the results via the FE simulation environment. Figure 1 shows the graphical abstract of the whole process in this paper, from the initial selection of the parameters to the extraction of optimal process parameters. It consists of three main steps, including mechanical part, machine learning part, optimisation and practical verification part. Initially, the important influenceable parameters that can affect the process are selected to be investigated, including pure cooling time, mould temperature, melt temperature, and pressure holding time. Then, the training dataset is produced by experimental design under FE analysis and SW Plastic. The results of defects in plastic products, including sink marks, shrinkage, and warpages are recorded. The produced dataset is modified in order to reach the highest efficiency using machine learning methods. The datasets are divided as 80% for training, 10% for validation, and another 10% for testing. The different machine learning methods including Decision tree (DT), multilayer perceptron (MLP), long short-term memory (LSTM), and gated recurrent units (GRU) models are trained, and their performances are investigated. Using the highest efficient methods the MOPSO is employed to extract the best process parameters to reach the lowest possible defects.

Section II provides an overview of the injection-moulding process for the intricate dashboard part. Additionally, this section elaborates on the approach for obtaining datasets using the finite element simulation environment. The methodology employed in this research, which integrates diverse soft computing methods and MOPSO, is elucidated in Section III. Section IV presents a comparative analysis and discussion of the outcomes extracted through the MATLAB-based developed model. The conclusions drawn from the study are summarized in Section V.

## Injection-moulding process for dashboard

Shrinkage, warpage, and sink marks are interconnected phenomena within injection moulding. The cooling and subsequent shrinkage of the material can induce uneven or differential contraction, resulting in deformation or warpage of the moulded part. Warpage denotes the departure from the intended shape of the part, leading to dimensional discrepancies and functional complications. On the other hand, sink marks can contribute to warpage by instigating stress concentrations due to uneven shrinkage, causing bending or twisting of the part. Consequently, the primary objective of this study is to minimize the occurrence of these identified defects by maintaining their values at the lowest possible levels.

**Figure 2:**
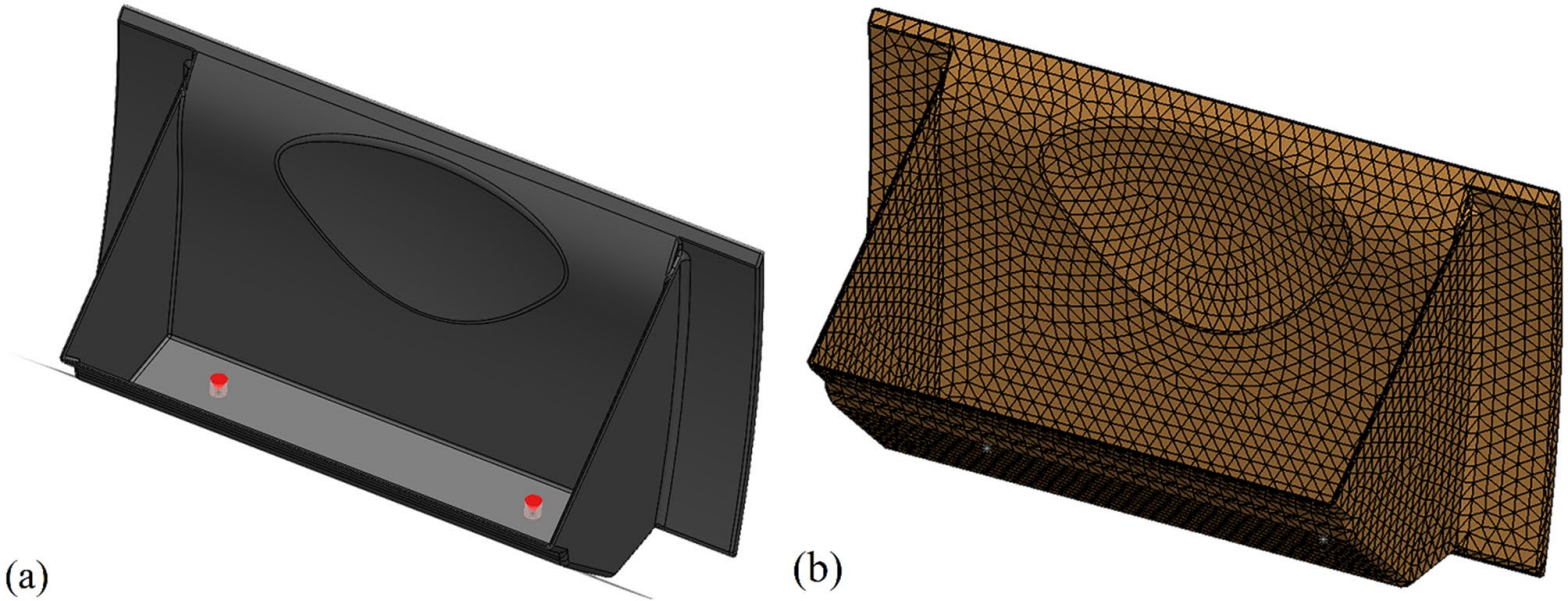
(**a**) Solid modelling of dashboard; (**b**) the meshed model with triangle meshes.

The literature survey identified three defects for in-depth analysis: warpage, shrinkage, and sink marks. A full factorial design is implemented to identify the most influential parameters that affect the chosen plastic part's quality to achieve an optimum design. Figure 2 illustrates the modelling of the dashboard, where (a) showcases the solid representation. The simulation process utilizes FE analysis and SW Plastic. The cooling system for the plastic part is developed utilizing a cool pipe model, wherein the cooling channels are incorporated into the solid representation of the mould design. In the iterative process, the transient thermal fields of the heated mould and cavity are computed by employing the Cool solver. For the initial heating, the ambient temperature is considered. Finite Element (FE) analysis plays a pivotal role in the simulation, ensuring the precision and accuracy of the analysis outcomes. Within Finite Element (FE) analysis, surface meshes employ triangle meshes that conform to the geometric characteristics of the samples, as exemplified in Fig. 2b. After evaluating various sizes, a surface mesh size of 1 mm is selected for the injection part.

This research investigates various process parameters, including pure cooling time, melt temperature, mould temperature, and pressure holding time. These parameters are examined at different levels, with 5 levels chosen for melt temperature as the most significant parameter. In comparison, 3 levels are selected for pure cooling time. These levels are selected through simulations to establish the minimum and maximum effective levels for each parameter and the required intermediary levels. Considering the chosen objectives and insights from the literature review, four key parameters are selected namely, cooling time, melt temperature, mould temperature, and pressure holding time. They are the most effective parameters for the evaluation of shrinkage, warpage, and sink mark^35^. For each parameter, the minimum and maximum levels are identified. Intermediate values are selected based on the equipment's capabilities, material characteristics, and desired results. Acrylonitrile Butadiene Styrene (ABS) is the designated material for this study. Considering the number of parameters and their corresponding levels outlined in Table 1, 180 simulations were conducted using SW Plastics.

**Table 1:** The selected parameters and their levels.

Parameters	L1	L2	L3	L4	L5
Melt temperature *T*_1_ (°C)	200	215	230	255	280
Mould temperature *T*_2_ (°C)	25	50	65	80	–
Pressure holding time *t*_1_ (s)	10	20	30	–	–
Pure cooling time *t*_2_ (s)	60	80	100	–	–

In addition, the theory of relaxation time of plastic materials provides insights into the dynamic behavior of plastics during cooling and solidification. By understanding and controlling factors that influence relaxation, such as cooling time, mold temperature, and material properties, manufacturers can minimize shrinkage, warpage, and sink marks in plastic products, resulting in higher quality end products.

Upon revisiting the methodology and considering the extensive literature on process parameters affecting defects in plastic products, the importance of parameters such as injection time, injection pressure, and holding pressure is acknowledged. While these parameters indeed play significant roles in the occurrence of defects like sink marks, shrinkage, and warpage, a focus is specifically placed on optimizing a subset of parameters for the chosen objectives. In selecting the parameters to study, the aim is to prioritize those with the most significant impact on the specific defects sought to be addressed: shrinkage, warpage, and sink marks. Based on the literature review and practical considerations, cooling time, melt temperature, mould temperature, and pressure holding time are identified as the key parameters directly influencing these defects. These parameters are chosen for their well-established relationships with shrinkage, warpage, and sink marks^35^. It is recognized that different studies enumerated several factors affecting in-mold shrinkage, including injection pressure, injection rate, and holding pressure. Additionally, the relationship between warpage and physical shrinkage is highlighted, particularly emphasizing the role of molding conditions such as melt temperature, pressure, and injection time in inducing nonuniform shrinkage. The decision to focus on cooling time, melt temperature, mould temperature, and pressure holding time is based on their direct influence on the specific defects under investigation and the practical constraints of the study.

## Methodology

**Figure 3:**
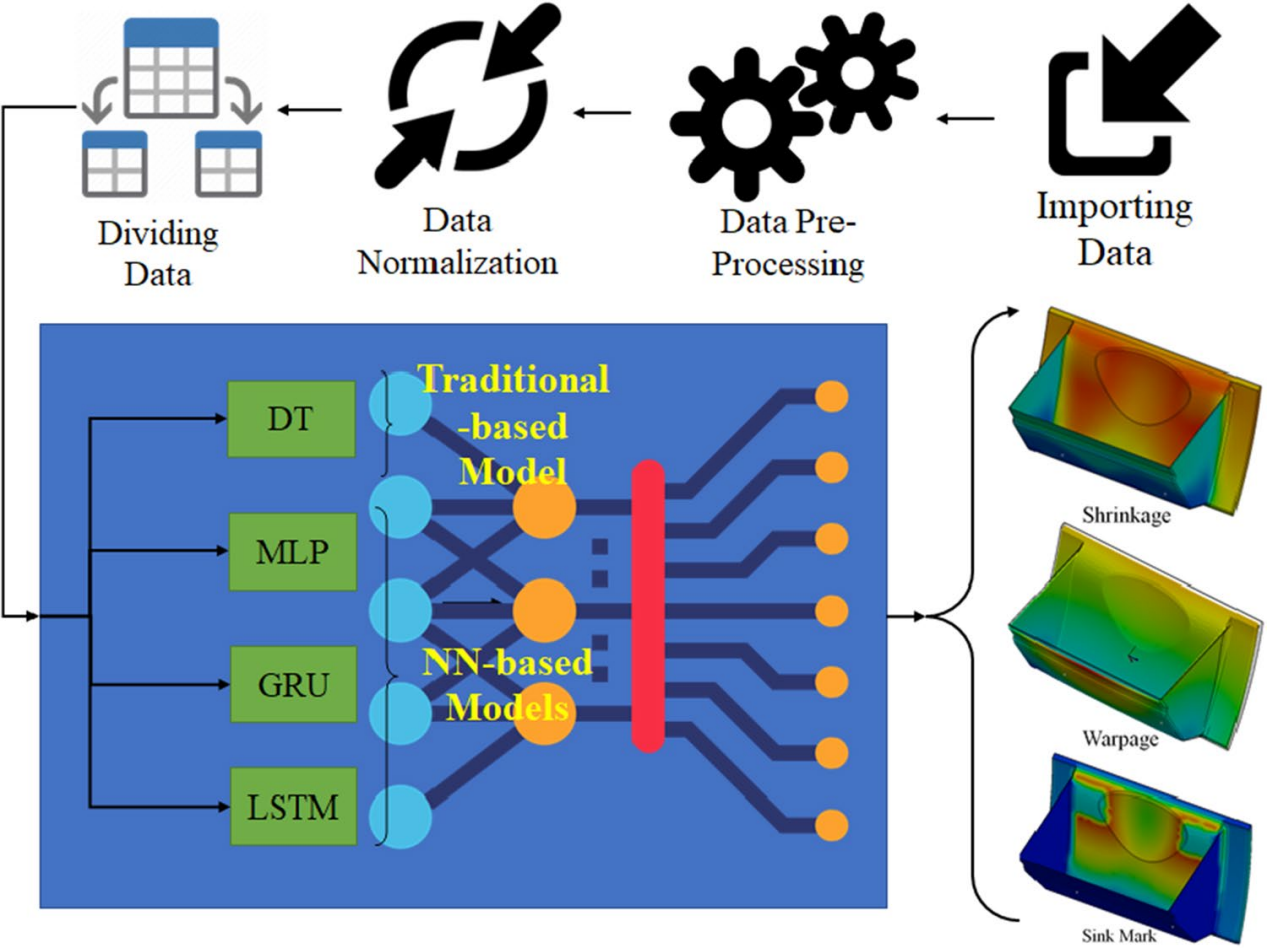
The schematic proposed hybrid soft computing algorithm in this study for calculation of the optimal process parameter of the injection moulding.

The primary aim of this study is to determine the optimal injection moulding process parameters for the dashboard to minimize warpage, shrinkage, and sink marks. The proposed method combines DT, MLP, LSTM and GRU soft computing. Figure 3 shows the schematic representation of the proposed technique from importing the dataset inside the algorithm until extraction of the maximum defects of the dashboard after injection. As the final step, this algorithm was used inside the cost function of the MOPSO to extract the optimal solutions. Each model has been trained individually, and then the combination of them is proposed as the most reliable model to calculate the defect of the final product.

### Data pre-processing

Before developing the model and utilizing the dataset, three essential tasks need to be carried out with the data. Firstly, any data outside the acceptable range should be excluded to enhance the system's resilience. The second task involves normalizing the data, which reduces complexity and prepares it for training. The normalized data can be obtained in the following manner:

1 $$n_{xi} = \frac{{x_{i} - \underline {x} }}{{\overline{x} - \underline {x} }}$$

Here, *n*_*xi*_ represents the normalized input data for the *i*th entry. The functions $\underline {x}$ and $\overline{x}$ are utilised to extract the minimum and maximum values from the dataset. To ensure realistic results, the data is divided into three groups during the final network pre-tuning process: 80% for training, 10% for validation, and another 10% for testing. The testing data is withheld from the network until the testing stage to achieve accurate and reliable outcomes using the proposed models.

### Decision tree

The first investigated model in this paper is DT. DT expands the data mining method with prominent usage in data analysis^36^. It can be used for classification and regression based on the criteria of the dataset. DT is composed of nodes and lines. There are two types of nodes, including leaf and branch nodes. The line represents the decision pathway between the parent and children. The decision boundaries are defined via the decision rule, an inequality expression. DT is employed for achieving many tasks in soft computing. The logic of the DT is to split data based on conditions and pass inputs to its children, called a binary tree. The described action is followed until the data reaches the leaf node as the final prediction results. Chi-squared automatic interaction detection, C4.5, and CART are the most common DT algorithm^37–39^. The privilege of the DT over other traditional prediction models is the ability to produce logical statements or interpretable rules. In this paper, the CART model, as a nonparametric regression, is employed based on recursive partitioning.

### Multilayer perceptron

The MLP is used for supervised training in classification and regression. Inputs and outputs adjust network parameters, minimizing error. Backpropagation derives optimal weights and biases from error measures like RMSE or MSE. Stochastic gradient descent updates biases and weights in the MLP. In the process of training, the output of the *j*th node for the *n*th data point can be denoted as:

2 $$e_{j} \left( n \right) = d_{j} \left( n \right) - y_{j} \left( n \right)$$

Given that *d* and *y* represent the target and actual outputs, respectively, the weights can be determined by minimizing the error through the utilization of the complete network output in the following manner:

3 $$\varepsilon \left( n \right) = \frac{1}{2}\sum\nolimits_{j} {e_{j}^{2} \left( n \right)}$$

The change in weights through the utilization of the gradient descent technique can be expressed as follows:

4 $$\Delta w_{ij} \left( n \right) = - \eta \frac{\partial \varepsilon \left( n \right)}{{\partial \upsilon_{i} \left( n \right)}}y_{i} \left( n \right)$$

where *y*_*i*_ represents the output of the previous neuron while $\upsilon_{i}$ denotes the local induced field. The learning rate *η* plays a crucial role in determining the MLP convergence. The Simplified version of the weight derivative based on the local induced field can be calculated for an output node in the following manner:

5 $$- \frac{\partial \varepsilon \left( n \right)}{{\partial \upsilon_{i} \left( n \right)}} = e_{j} \left( n \right)\phi^{\prime } \left( {\upsilon_{j} \left( n \right)} \right)$$

The weight derivative of the hidden node can be extracted as the activation function derivative, denoted as *ϕ*′, which remains unchanged with respect to the weight and locally induced field. This can be mathematically represented as follows:

6 $$- \frac{\partial \varepsilon \left( n \right)}{{\partial \upsilon_{i} \left( n \right)}} = \phi^{\prime } \left( {\upsilon_{j} \left( n \right)} \right)\sum\nolimits_{k} { - \frac{\partial \varepsilon \left( n \right)}{{\partial \upsilon_{k} \left( n \right)}}w_{kj} \left( n \right)}$$

The modification in weight for the hidden node is determined by the weight of the *k*th node in the output layer, as indicated by Eq. (6). Consequently, the weights in the output layer are affected by the weights in the hidden layer, which is determined by the activation function derivative. This mechanism is commonly referred to as the activation function backpropagation^40^.

### Long-short term memory

The RNN is a useful approach for forecasting nonlinear signals, as it accounts for the dynamic nature of complicated mechanical phenomena. Its internal memory blocks enable the learning of temporal sequences. However, RNN has limitations, including strict time lag dependence and the inability to capture long-term dependencies. LSTM overcomes these drawbacks and can handle extended sequences of data samples. It incorporates three gates: the input gate, output gate, and forget gate, which preserve gradient information and regulate the flow of information. Fully connected regression layers generate the output of the LSTM. Memory cells establish connections and retain temporal states to determine the flow of information.

The input and output sequences of the model are represented as ${\mathbf{x}} = \left( {x_{1} ,x_{2} , \ldots ,x_{T} } \right)$ and $m = \left( {m_{1} ,m_{2} , \ldots ,m_{T} } \right)$, respectively, where *T* corresponds to the prediction period. The memory cell for the *j*th neuron at time t is referred to as $c_{t}^{j}$. The output of the *j*th neuron, denoted as $m_{t}^{j}$, is given by:

7 $$m_{t}^{j} = o_{t}^{j} \tanh \left( {c_{t}^{j} } \right)$$

The output gate, $o_{t}^{j}$, is responsible for determining which information should be propagated. The expression for the output gate can be given as:

8 $$o_{t}^{j} = \sigma \left( {W_{o} x_{t} + U_{o} m_{t - 1} - V_{o} c_{t} } \right)^{j}$$

The vector representations of $m_{t - 1}^{j}$ and $c_{t}^{j}$ are represented by $m_{t - 1}$ and *c*_*t*_, respectively. Diagonal weight matrices *W*_*o*_, *U*_*o*_, and *V*_*o*_ play a role in minimizing a loss function and require online tuning. Additionally, the ${\varvec{\sigma}}$ represents a standard logistic sigmoid function. The recurrent CEC unit serves as the primary focus of the memory cell, responsible for generating the cell state. At each time step, the memory cell, $c_{t}^{j}$, needs to be updated. This update involves eliminating the current memory cell and incorporating the new memory value, $\tilde{c}_{t}^{j}$, using the following:

9 $$c_{t}^{j} = f_{t}^{j} c_{t - 1}^{j} + i_{t}^{j} \tilde{c}_{t}^{j}$$

where the new memory value is:

10 $$\tilde{c}_{t}^{j} = \tanh \left( {W_{c} x_{t} + U_{c} m_{t - 1} } \right)^{j}$$

A forget gate is utilised to ensure that the internal cell values do not exhibit unbounded growth and to maintain the continuous operation of the time series mechanism instead of segmentation. This gate enables the reset of outdated information flow. It replaces the CEC weight with the activation of the multiplicative forget gate. The computation of the forget gate, denoted as $f_{t}^{j}$, is performed after updating the memory cell with the new memory value according to the following equation:

11 $$f_{t}^{j} = \sigma \left( {W_{f} x_{t} + U_{f} h_{t - 1} + V_{f} c_{t - 1} } \right)^{j}$$

The diagonal weight matrices *W*_*f*_, *U*_*f*_, and *V*_*f*_ are utilized in the computation of the forget gate. Similarly, the input gate follows the same methodology to determine the reserved new features, expressed as:

12 $$i_{t}^{j} = \sigma \left( {W_{i} x_{t} + U_{i} h_{t - 1} + V_{i} c_{t - 1} } \right)^{j}$$

The diagonal weight matrices *W*_*i*_, *U*_*i*_, and *V*_*i*_ are involved in this computation as well. It is important to note that the values of the three gates fall between 0 and 1. The LSTM output can be formulated as follows:

13 $$y = g\left( {W_{d} h_{t} + b_{d} } \right)$$

The function *g* is in the range of [− 2,2] as a centred logistic sigmoid function. The training process of LSTM involves backpropagation through time and real-time recurrent learning, utilizing gradient descent optimisation. The loss function is employed as the sum of square errors. LSTM leverages the linear CEC of the memory cell to mitigate errors and handle extended prediction horizons.

### Gated recurrent unit

A GRU layer learns dependencies in time series data. The hidden state at each step contains the layer's output. Gates control information updates. Weights include input weights (*W*), recurrent weights (*R*), and bias (*b*). Additional bias values are needed for certain gate and state calculations. Matrices *W* and *R* are input and recurrent weights concatenations, respectively. These matrices are concatenated as follows:

14 $${\mathbf{W}} = \left[ {\begin{array}{*{20}c} {W_{r} } \\ {W_{z} } \\ {W_{{\hat{h}}} } \\ \end{array} } \right]$$

15 $${\mathbf{R}} = \left[ {\begin{array}{*{20}c} {R_{r} } \\ {R_{z} } \\ {R_{{\hat{h}}} } \\ \end{array} } \right]$$

The representation of the reset gate, update gate, and candidate state is given by *r*, *z*, and $\hat{h}$, respectively. The configuration of the bias vector depends on the ResetGateMode property. In the case of ResetGateMode being 'after-multiplication' or 'before-multiplication', the bias vector is constructed by combining three separate vectors:

16 $${\mathbf{b}} = \left[ {\begin{array}{*{20}c} {b_{{W_{r} }} } \\ {b_{{W_{z} }} } \\ {b_{{W_{{\hat{h}}} }} } \\ \end{array} } \right]$$

The subscript *W* denotes that this bias corresponds to the multiplication with input weights. On the other hand, if the ResetGateMode is set to 'recurrent-bias-after-multiplication', the bias vector is created by combining six distinct vectors:

17 $${\mathbf{b}} = \left[ {\begin{array}{*{20}c} {\begin{array}{*{20}c} {b_{{W_{r} }} } \\ {b_{{W_{z} }} } \\ {b_{{W_{{\hat{h}}} }} } \\ \end{array} } \\ {\begin{array}{*{20}c} {b_{{R_{r} }} } \\ {b_{{R_{z} }} } \\ {b_{{R_{{\hat{h}}} }} } \\ \end{array} } \\ \end{array} } \right]$$

The subscript *R* indicates that this bias corresponds to the multiplication with recurrent weights. The hidden state at time step t is calculated as:

18 $${\mathbf{h}}_{{\mathbf{t}}} = \left( {1 - {\mathbf{z}}_{{\mathbf{t}}} } \right) \odot {\hat{\mathbf{h}}}_{{\mathbf{t}}} + {\mathbf{z}}_{{\mathbf{t}}} \odot {\hat{\mathbf{h}}}_{{{\mathbf{t - 1}}}}$$

The following formulas describe the components at time step t.

19 $$r_{t} = \sigma_{g} \left( {W_{r} x_{t} + bW_{r} + R_{r} h_{t - 1} } \right)$$

20 $$r_{t} = \sigma_{g} \left( {W_{r} x_{t} + bW_{r} + R_{r} h_{t - 1} + bR_{r} } \right)$$

21 $$z_{t} = \sigma_{g} \left( {W_{z} x_{t} + bW_{z} + R_{z} h_{t - 1} } \right)$$

22 $$z_{t} = \sigma_{g} \left( {W_{z} x_{t} + bW_{z} + R_{z} h_{t - 1} + bR_{z} } \right)$$

23 $$\hat{h}_{t} = \sigma_{s} \left( {W_{{\hat{h}}} x_{t} + bW_{{\hat{h}}} + r_{t} \odot \left( {R_{{\hat{h}}} h_{t - 1} } \right)} \right)$$

24 $$\hat{h}_{t} = \sigma_{s} \left( {W_{{\hat{h}}} x_{t} + bW_{{\hat{h}}} + R_{{\hat{h}}} \odot \left( {r_{t} h_{t - 1} } \right)} \right)$$

25 $$\hat{h}_{t} = \sigma_{s} \left( {W_{{\hat{h}}} x_{t} + bW_{{\hat{h}}} + R_{{\hat{h}}} \odot \left( {R_{{\hat{h}}} h_{t - 1} + bR_{z} } \right)} \right)$$

During these computations, the activation functions for the gate and state are represented as *σ*_*g*_ and *σ*_*s*_, respectively.

The default behaviour of the GRU Layer function involves using the sigmoid function, defined as $\sigma \left( s \right) = \left( {1 + e^{ - x} } \right)^{ - 1}$, to calculate the gate activation function. The hyperbolic tangent function (tanh) is also employed to compute the state activation function. The StateActivationFunction and GateActivationFunction properties can be modified to customize the state and gate activation functions.

### Multiple objectives particle swarm optimisation

PSO has been widely used for optimisation problems since its proposal by Kennedy and Eberhart in 1997^41^. To address MOO problems, an extended version called MOPSO was introduced by Coello et al. in 2004^42^. MOPSO incorporates an external repository, consisting of an archive controller and an adaptive grid, to store the non-dominated solutions discovered during the search process.

The archive controller determines whether a new solution should be added to or removed from the archive. In comparison, the adaptive grid aims to distribute the objective function space uniformly by dividing it into regions. In contrast, traditional methods convert multiple objectives into a single objective. MOPSO directly handles MOO problems without needing multiple runs and achieves more accurate results, especially for disjointed and concave Pareto fronts. However, in MOO problems, multiple global optima exist along the Pareto front. Therefore, MOPSO maintains a repository of nondominated particles and employs the adaptive grid method to assign each particle a leader from the repository. This approach ensures a diverse and accurate approximation of the Pareto front.

In this research, the provided platform in MATLAB by Víctor Martínez-Cagigal^43^ is employed to extract the 3-dimensional Pareto front distribution of the optimal injection moulding process parameters. In order to vectorize the objective function for our case, the three different defects, including sink marks, shrinkage, and warpages are considered simultaneously in order to find the pareto front optimal solutions of the process. However, the operator can choose the suitable option between the recommended optimal solutions from the extracted data.

## Results and discussions

This Section is composed of two subsections. In the first subsection, we focus on the soft computing modelling of the system in order to extract the most reliable models to calculate the sink marks, shrinkage, and warpage based on melt temperature, mould temperature, pressure holding time, and pressure cooling time. Four investigated methods in previous Sections are investigated in this matter. In the following subsections, the most accurate extracted soft computing method in calculating sink marks, shrinkage, and warpage is employed inside the multiple objectives' optimisation (based on subsection III.F) to extract the optimal solution of the operation in order to minimise the defects of the final product. At last, some of the extracted optimal solutions via MOPSO are investigated to prove the efficiency of the proposed method. Five parameters have been used for validation of our investigated methods in order to choose the most reliable one, including correlation coefficient (CC), mean square error (MSE), root mean square error (RMSE), and normalized root mean square error (NRMSE). These validation parameters are calculated as follows:

26 $${\text{CC}} = \frac{{\sum\nolimits_{i = 1}^{n} {\left( {x_{i} - \overline{x}} \right)\left( {T_{i} - \overline{T}} \right)} }}{{\sqrt {\sum\nolimits_{i = 1}^{n} {\left( {x_{i} - \overline{x}} \right)^{2} \left( {T_{i} - \overline{T}} \right)^{2} } } }}$$

27 $${\text{MSE}} = \frac{1}{n}\sum\limits_{i = 1}^{n} {\left( {T_{i} - \overline{T}} \right)}^{2}$$

28 $${\text{RMSE}} = \sqrt {\frac{{\sum\nolimits_{i = 1}^{n} {\left( {T_{i} - \hat{T}_{i} } \right)^{2} } }}{n}}$$

29 $${\text{NRMSE}} = \frac{{{\text{RMSE}}}}{{\overline{T}}}$$

where *n*, *x*_*i*_, $\overline{x}$ and $\overline{T}$ are the number of samples, the *i*th input, the mean of inputs and the mean of outputs.

### Modelling

Four different soft computing methods are investigated in subsection III.B-D. DT, MLP, GRU, and LSTM are designed and developed under MATLAB software using functions including fitrtree, feedforwardnet, gru, and lstm. As there are three different defects, including sink marks, shrinkage, and warpage, three different soft computing functions are needed to calculate these parameters based on T1, T2, t1, and t2. The DT, MLP, GRU, and LSTM hyperparameters are extracted using trial and error to reach the most efficient model for each of them, shown in Table 2. The four investigated models are trained using 80% of the datasets. In addition, the remaining dataset (20%) is used for testing the models to pick the most appropriate one for each parameter. Table 3 shows the outcomes of this process for all four investigated methods in cooperation with three outputs (sink marks, shrinkage, and warpage). Based on the represented results in Table 3, MLP is the best model to imitate the system's behaviour for calculating warpage and shrinkage. In addition, DT is the best option for calculating the sink mark defect based on four investigated inputs of the system. These extracted models are called shrinkage_MLP, warpage_MLP, and sinkmark_DT. The outputs are visualised using the plot function of MATLAB software.

**Table 2:** The extracted most efficient hyperparameters for four investigated soft computing methods (DT, MLP, GRU, and LSTM).

Methods	Hyperparameters
DT	Categorical predictors = 3; ' minimum parent size = 4; maximum number of splits = 29; surrogate = 'on
MLP	Number of hidden layer = 1; number of neurons = 4; training function = 'trainscg'; mutation = 8.38 × 10^−3^
GRU	Number of hidden layer = 1; number of neurons = 10; maximum epochs = 500; minimum batch size = 32; learning function = ‘adam’; learning rate = 7.58 × 10^−3^; dropout value = 0.5
LSTM	Number of hidden layer = 1; number of neurons = 8; maximum epochs = 500; minimum batch size = 32; learning function = ‘adam’; learning rate = 9.18 × 10^−3^; dropout value = 0.5

**Table 3:** The extracted results for investigation of implementing DT, MLP, GRU, and LSTM for calculation of sink marks, shrinkage, and warpage based on melt temperature, mould temperature, pressure holding time, and pressure cooling time.

Index	CC			MSE			RMSE			NRMSE		
	Train	Test	All	Train	Test	All	Train	Test	All	Train	Test	All
*Shrinkage*												
DT	0.9777	0.9584	0.9741	9.4 × 10^−2^	1.6 × 10^−1^	1.1 × 10^−1^	3.1 × 10^−1^	4.0 × 10^−1^	3.3 × 10^−1^	4.1 × 10^−2^	5.3 × 10^−2^	4.4 × 10^−2^
**MLP**	**0.9657**	**0.9458**	**0.9599**	**6.0 × 10**^**−7**^	**8.9 × 10**^**−7**^	**6.6 × 10**^**−7**^	**7.7 × 10**^**−4**^	**9.5 × 10**^**−4**^	**8.1 × 10**^**−4**^	**3.8 × 10**^**−2**^	**4.6 × 10**^**−2**^	**4.0 × 10**^**−2**^
GRU	0.6639	0.5519	0.6431	1.45	1.50	1.46	1.21	1.23	1.21	0.16	0.16	0.16
LSTM	0.4997	0.4156	0.4839	1.60	1.62	1.61	1.27	1.27	1.27	0.17	0.17	0.17
*Warpage*												
DT	0.9762	0.9258	0.9638	6.4 × 10^−2^	2.7 × 10^−1^	1.0 × 10^−1^	2.5 × 10^−1^	5.1 × 10^−1^	3.2 × 10^−1^	3.6 × 10^−2^	7.3 × 10^−2^	4.6 × 10^−2^
**MLP**	**0.9895**	**0.9635**	**0.9847**	**4.6 × 10**^**−2**^	**1.2 × 10**^**−1**^	**6.1 × 10**^**−2**^	**2.1 × 10**^**−1**^	**3.5 × 10**^**−1**^	**2.5 × 10**^**−1**^	**3.1 × 10**^**−2**^	**4.9 × 10**^**−2**^	**3.5 × 10**^**−2**^
GRU	0.8610	0.8441	0.8599	6.5 × 10^−1^	8.8 × 10^−1^	7.0 × 10^−1^	8.1 × 10^−1^	9.4 × 10^−1^	8.4 × 10^−1^	1.2 × 10^−1^	1.3 × 10^−1^	1.2 × 10^−1^
LSTM	0.8001	0.8037	0.8015	7.8 × 10^−1^	9.1 × 10^−1^	8.0 × 10^−1^	8.8 × 10^−1^	9.5 × 10^−1^	9.0 × 10^−1^	1.3 × 10^−1^	1.3 × 10^−1^	1.3 × 10^−1^
*Sink Mark*												
**DT**	**0.9979**	**0.9873**	**0.9958**	**2.1 × 10**^**−8**^	**6.3 × 10**^**−8**^	**3.0 × 10**^**−8**^	**1.5 × 10**^**−4**^	**2.5 × 10**^**−4**^	**1.7 × 10**^**−4**^	**7.2 × 10**^**−3**^	**1.2 × 10**^**−2**^	**8.5 × 10**^**−3**^
MLP	0.9657	0.9458	0.9599	6.0 × 10^−7^	8.9 × 10^−7^	6.6 × 10^−7^	7.7 × 10^−4^	9.5 × 10^−4^	8.1 × 10^−4^	3.8 × 10^−2^	4.6 × 10^−2^	4.0 × 10^−2^
GRU	0.9573	0.9705	0.9616	6.6 × 10^−7^	5.1 × 10^−7^	6.3 × 10^−7^	8.1 × 10^−4^	7.2 × 10^−4^	7.9 × 10^−4^	4.0 × 10^−2^	3.5 × 10^−2^	3.9 × 10^−2^
LSTM	0.9681	0.9853	0.9727	7.4 × 10^−7^	4.8 × 10^−7^	6.9 × 10^−7^	8.6 × 10^−4^	6.9 × 10^−4^	8.3 × 10^−4^	4.2 × 10^−2^	3.4 × 10^−2^	4.1 × 10^−2^

Significant values are in bold.

**Figure 4:**
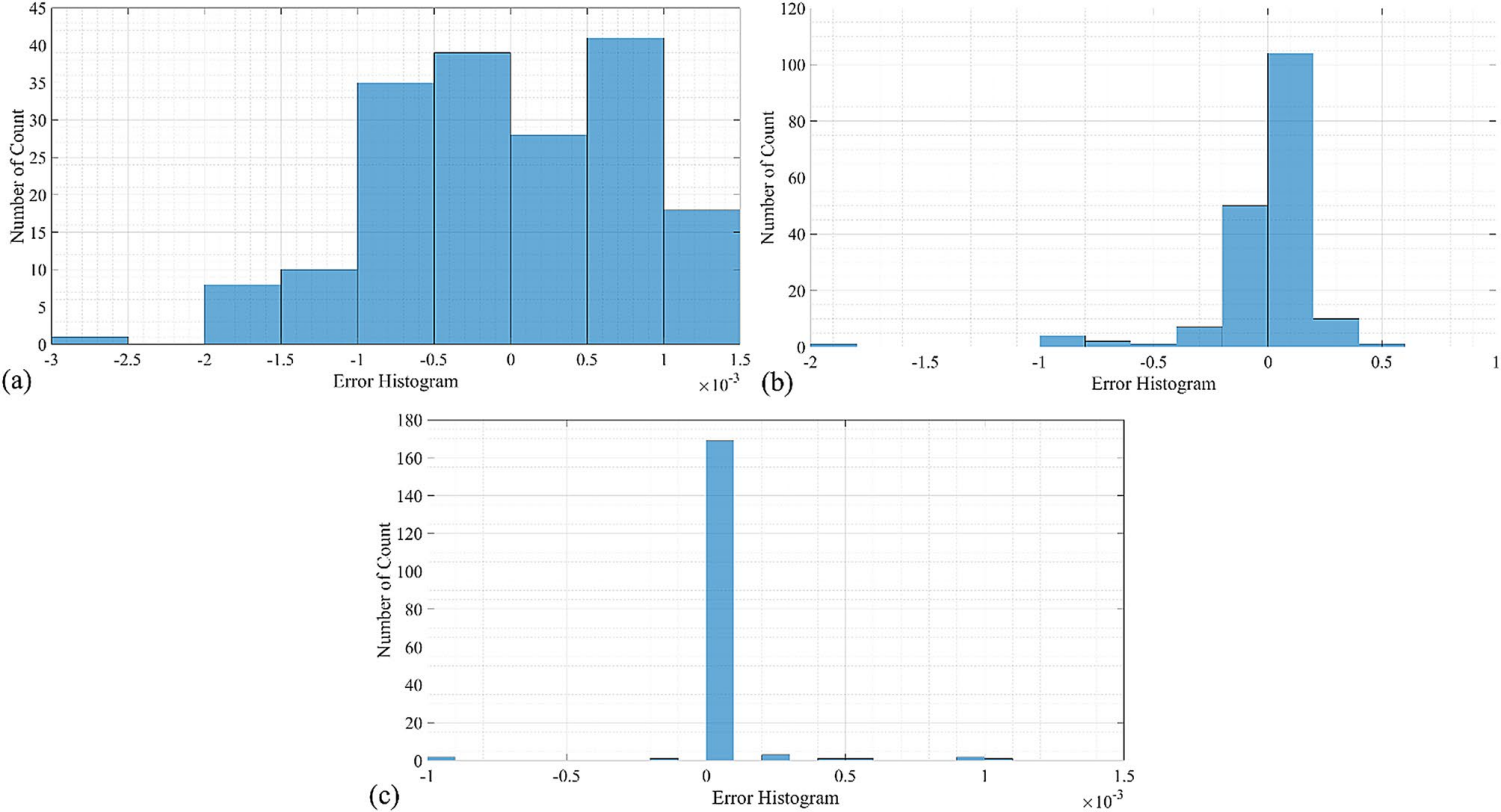
The error histogram of the data using all data, including all data (training and testing) using (**a**) shrinkage_MLP; (**b**) warpage_MLP; (**c**) sinkmark_DT.

**Figure 5:**
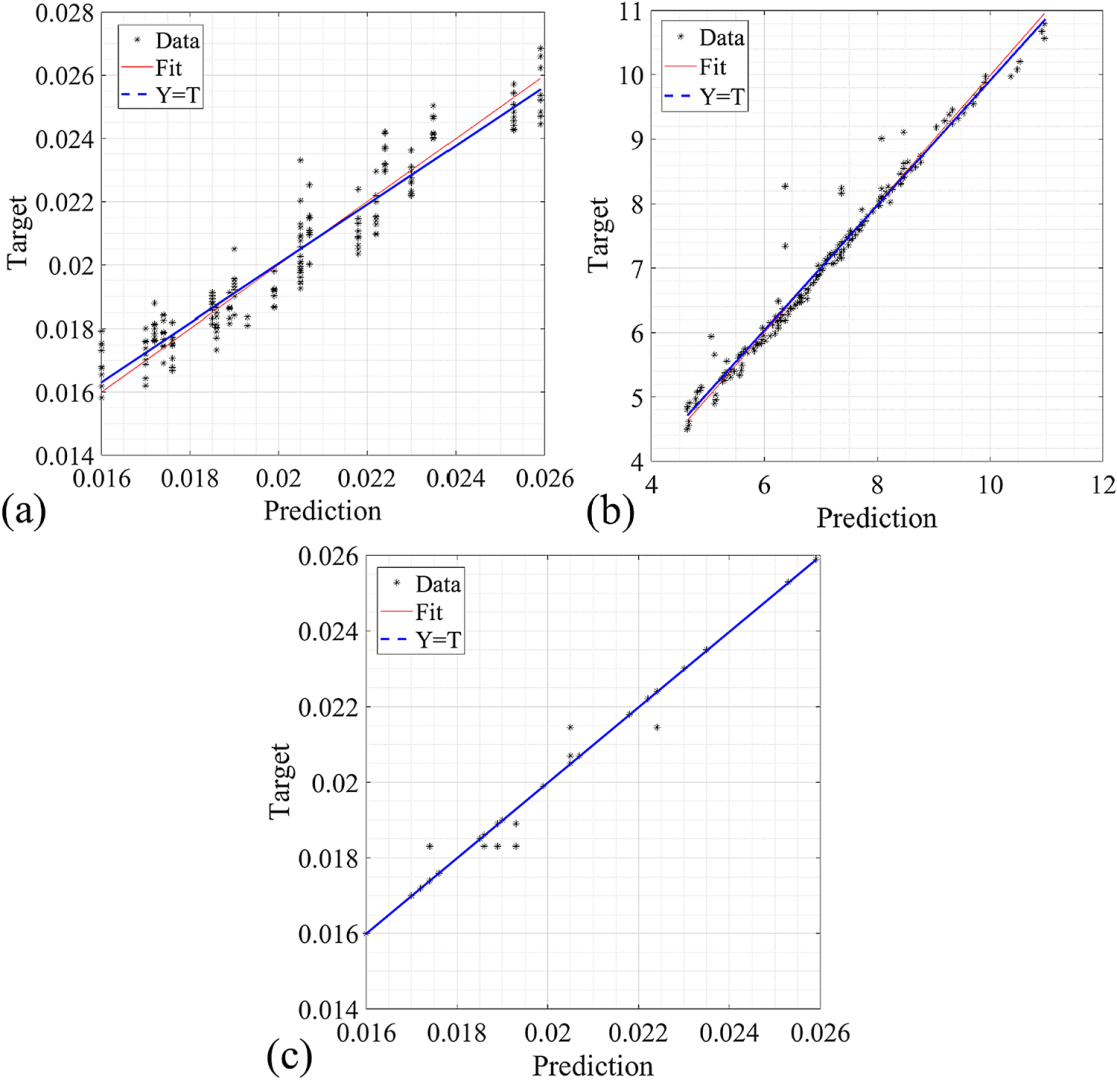
The regression of the data during all data (training and testing) using optimized based (**a**) shrinkage_MLP; (**b**) warpage_MLP; (**c**) sinkmark_DT.

**Figure 6:**
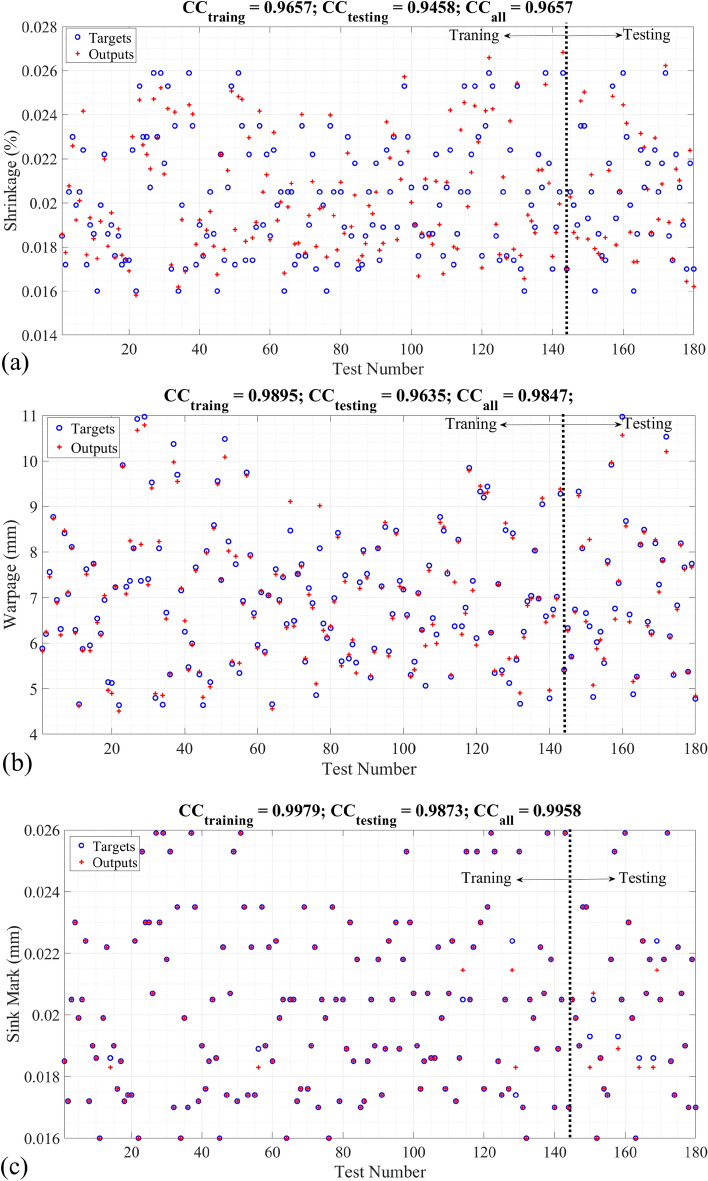
The actual and predicted defects of injection-moulding process of dashboard using (**a**) shrinkage_MLP; (**b**) warpage_MLP; (**c**) sinkmark_DT.

The graphical representations of Table 3 are shown in the following Figs. 4, 5 and 6. The error histogram of these three extracted models, including shrinkage_MLP, warpage_MLP, and sinkmark_DT using all datasets (training 80% and testing 20%) are shown in Fig. 4. Figure 4a shows the error histogram of the shrinkage_MLP with mean and standard deviation of error of are 2.28 × 10^−5^ and 8.14 × 10^−4^, respectively. It shows the left-skewed histogram behaviour. Figure 4b shows the error histogram of the warpage_MLP with mean and standard deviation of error of − 1.21 × 10^−3^ and 2.48 × 10^−1^, respectively. It shows the left-skewed histogram behaviour. Figure 4c shows the error histogram of the sinkmark_DT with mean and standard deviation of error of 1.53 × 10^−5^ and 1.72 × 10^−4^, respectively. It shows the normal distribution of the error as DT is the traditional soft computing method.

Figure 5a–c represents the regression of the developed shrinkage_MLP, warpage_MLP and sinkmark_DT for predicting the algorithms' injection-moulding defects during all datasets (training and testing stages), respectively. The vertical and horizontal axes in Fig. 5 represent the actual and predicted defect (including shrinkage, warpage and sink mark). Based on Fig. 5a–c, the R-square of shrinkage_MLP, warpage_MLP and sinkmark_DT are 0.9074, 0.9713 and 0.9961, respectively (Fig. 5a–c).

Figure 6a–c represents the target and predicted shrinkage, warpage, and sink mark of the dashboard injection moulding product based on T1, T2, t1, and t2 using shrinkage_MLP, warpage_MLP and sinkmark_DT, respectively. Following the outcomes in Fig. 6a, the CC linking the target and predicted shrinkage correspondingly stands at 0.9657, 0.9458, and 0.9657 for the training, testing, and all datasets. Turning our attention to the findings in Fig. 6b, the CC connecting the target and predicted warpage are appraised at 0.9895, 0.9635, and 0.9847 during the training, testing, and all dataset analyses, respectively. The information divulged by Fig. 6c unfolded a similar pattern, with the CC bridging the gap between the target and predicted sink mark measuring at 0.9979, 0.9873, and 0.9958 for the training, testing, and all datasets in sequence.

The extracted and investigated models in this subsection are employed inside the MOPSO in the next subsection to extract the optimal solutions of the process.

Reducing the cooling time minimizes the duration the plastic remains in the mold, thereby decreasing the likelihood of uneven cooling and reducing residual stresses. Consequently, this diminishes the occurrence of warpage and sink marks. Furthermore, lowering the melt temperature alleviates thermal stress on the molded part, resulting in more uniform cooling and solidification, thereby reducing shrinkage and warpage. Additionally, decreasing the pressure holding time prevents excessive material packing, which can cause sink marks and internal stresses. Striking a balance ensures the material is adequately packed without being overly compressed. Finally, elevating the mold temperature fosters a uniform cooling rate across the part, mitigating differential shrinkage and warpage while enhancing material flow and packing, thus minimizing sink marks.

In the study, the focus is on optimizing cooling time to minimize the duration that the plastic remains in the mold. By reducing the cooling time, the aim is to mitigate the potential for uneven cooling and residual stresses, which are known contributors to warpage, shrinkage, and sink marks. The rationale for emphasizing low cooling time parameters was based on the understanding that prolonged exposure to the mold can exacerbate these defects.

In the study, one of the focus is on optimizing cooling time to minimize the duration that the plastic remains in the mold. By reducing the cooling time, the aim is to mitigate the potential for uneven cooling and residual stresses, which are known contributors to warpage, shrinkage, and sink marks. The rationale for emphasizing low cooling time parameters was based on the understanding that prolonged exposure to the mold can exacerbate these defects.

The relaxation time of plastic materials is also pivotal in determining shrinkage, warpage, and sink marks in molded products. During molding processes, plastic undergoes stress from shaping and cooling. Subsequently, as stress dissipates, the material relaxes over time, regulated by its relaxation time. This phenomenon significantly affects the extent and uniformity of shrinkage. Insufficient relaxation time may result in residual stresses, causing uneven shrinkage and dimensional inaccuracies. Uneven stress relaxation can lead to differential shrinkage and warpage. Sink marks, stemming from localized shrinkage during cooling, are also influenced by relaxation time. Inadequate relaxation time may prevent uniform stress redistribution, resulting in localized areas of shrinkage and sink marks. Overall, controlling relaxation time is vital for ensuring dimensional stability and surface quality in plastic components, mitigating defects such as shrinkage, warpage, and sink marks.

### Optimisation

The three investigated models (shrinkage_MLP, warpage_MLP and sinkmark_DT) are the objective functions of the multiple objectives’ optimisation using PSO, investigated in subsection III.F. As mentioned before, the provided toolbox by Víctor Martínez-Cagigal^43^ is used for the implementation purpose of this study.

**Figure 7:**
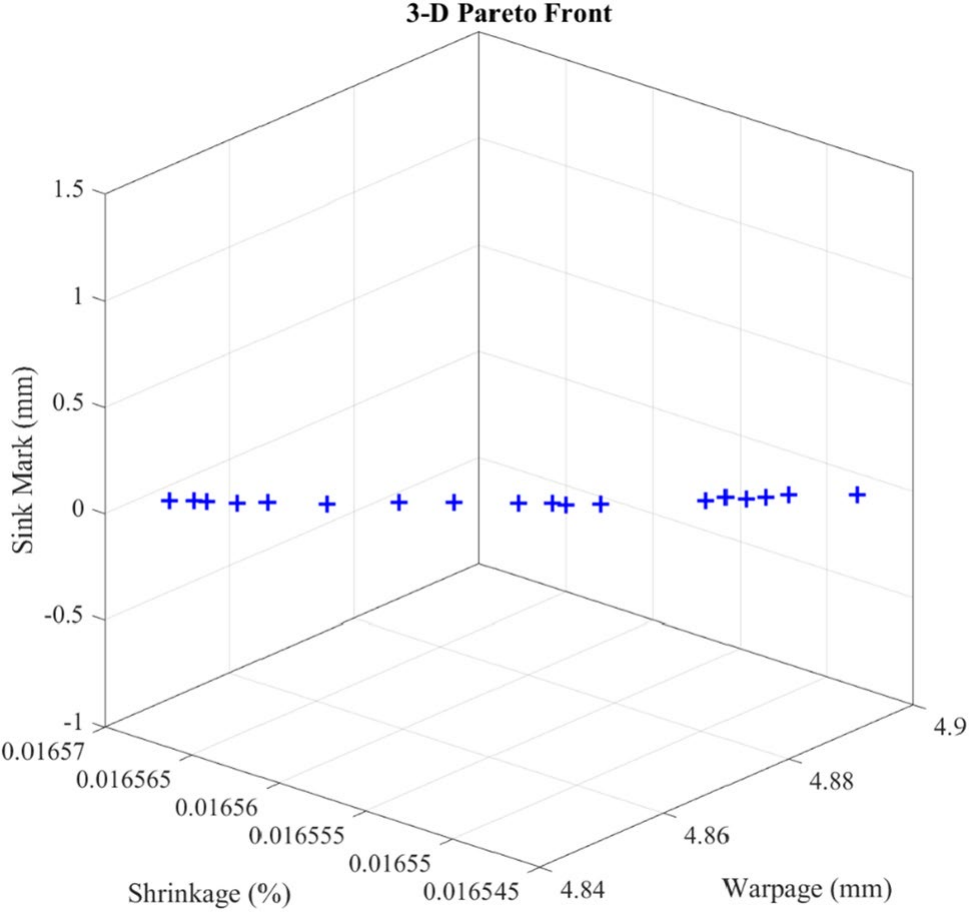
Three-dimensional Pareto front of the extracted optimal solution using MOPSO in injection-moulding process to calculate the minimal defects. Briefly, the MLP and DT soft computing methods are used to calculate the injection-moulding defects of dashboard products based on the process parameters (T1, T2, t1, and t2). MOPSO calculates the optimal process parameters to lead to the minimum defect of the final product.

Illustrated in Fig. 7 is the distribution of the optimal solution's three-dimensional Pareto front configuration within the context of the process. Moreover, the optimal solutions derived with extracted defects are outlined in Table 4. A sequence of injection-moulding experiments was conducted to affirm the system's precision using the extracted optimal solution from the recently introduced approach. The extracted optimal solution results in terms of shrinkage, warpage and sink marks using the optimal machine learning methods and SW Plastics are shown inside the Table 4. Also, the margin of error between the experiment and predicted values via investigated soft computing methods is mentioned in the last column of Table 4 as the average of three defects error between machine learning methods and SW Plastics. The process of determining the margin of error between the experiments detailed in Table 4 and the optimization methods involves comparing the predicted values acquired from the experiments with the optimized values derived from the optimization method. This comparison enables the identification of differences among the chosen approaches.

**Table 4:** Extracted optimal process parameters of injection-moulding process of dashboard using soft computing methods and MOPSO with extracted defects, including shrinkage, warpage, and sink mark.

No	T1	T2	t1	t2	Shrinkage		Warpage		Sink mark		Error %
					MLP	SW Plastic	MLP	SW Plastic	DT	SW Plastic	
1	203.911	75.071	12.478	78.471	0.0165	0.0181	4.886	4.4951	0.016	0.0174	9
2	203.912	75.065	12.452	78.475	0.0166	0.0151	4.885	4.1522	0.016	0.0179	13
3	203.913	75.073	12.109	78.484	0.0166	0.0199	4.870	4.3830	0.016	0.0141	14
4	203.912	75.074	11.673	78.482	0.0166	0.0183	4.849	4.2186	0.016	0.0125	15
5	203.914	75.068	12.018	78.474	0.0166	0.0159	4.866	5.2553	0.016	0.017	6
6	203.910	75.074	12.679	78.473	0.0165	0.0185	4.895	5.5313	0.016	0.0133	14
7	203.912	75.068	11.938	78.480	0.0166	0.0148	4.862	4.0841	0.016	0.0179	13
8	203.912	75.069	12.517	78.474	0.0165	0.0168	4.888	4.6925	0.016	0.0165	3
9	203.911	75.076	11.710	78.480	0.0166	0.0168	4.851	4.7055	0.016	0.0157	2
10	203.911	75.072	11.752	78.479	0.0166	0.0154	4.853	4.3192	0.016	0.0179	10
11	203.913	75.0759	12.177	78.479	0.0166	0.0129	4.873	5.5065	0.016	0.0195	19
12	203.911	75.074	11.626	78.489	0.0166	0.0178	4.846	4.4583	0.016	0.0170	7
13	203.911	75.074	11.831	78.478	0.0166	0.0148	4.856	5.1959	0.016	0.0150	12
14	203.913	75.065	12.564	78.475	0.0165	0.0163	4.890	4.9878	0.016	0.0152	4
15	203.910	75.0723	12.407	78.478	0.0166	0.0188	4.883	5.7619	0.016	0.0182	15
16	203.911	75.070	11.658	78.474	0.0166	0.0158	4.848	4.7026	0.016	0.0171	5
17	203.911	75.074	12.233	78.483	0.0166	0.0178	4.875	4.1924	0.016	0.0174	10
18	203.912	75.071	12.160	78.475	0.0166	0.0188	4.872	4.3361	0.016	0.0189	14

## Conclusion

This study focuses on assessing and mitigating defects in plastic products through injection moulding. Key process parameters were meticulously examined, including pure cooling time, mould temperature, melt temperature, and pressure holding time, using a full factorial design of experiments. These parameters significantly influence the final product's physical and mechanical properties. By employing soft computing techniques like FE analysis, the study effectively quantified and mitigated the impact of various input parameters on the injection moulding process. CAD models integrated with FE simulations facilitated the assessment of shrinkage, warpage, and sink marks. Among various models, including DT, MLP, GRU and LSTM, the best-performing model was identified for defect prediction. Furthermore, the application of MOPSO extracted optimal process parameters. The proposed method, implemented in MATLAB, yielded 18 optimal solutions based on the Pareto-Front. For practitioners, this study highlights the significance of process parameters in enhancing product quality. It introduces effective tools for defect prediction and optimization. In conclusion, this research provides insights into optimizing injection moulding processes, ultimately improving product quality and manufacturing efficiency. Future research should focus on advanced soft computing techniques such as unsupervised deep learning methods, real-time optimization, robustness against uncertainties, integration with Industry 4.0, and sustainability in injection moulding. Also, reinforcement learning can be used for dynamic multi-objective optimization to relocate the PSO more adaptively (Supplementary Information).

## Supplementary Information

Supplementary Information.

## Data Availability

The datasets analysed during the current study are available for the corresponding author on reasonable request.

## Abbreviations

ANOVA: Analysis of variance
CART: Classification and regression trees
CEC: Constant error carousel
DT: Decision tree
FE: Finite element
FIS: Fuzzy inference system
GA: Genetic algorithm
GCNN: Guided convolutional neural network
GRU: Gated recurrent units
KBS: Knowledge-based system
LSTM: Long-short term memory
MLP: Multilayer perceptron
MOO: Multi-objective optimisation
MSE: Mean square error
QSSPP: Quality-relevant step-wise sequential phase partition
RNN: Recurrent neural network
SVR: Support vector regression
