# Supplementary material for: Multiple objectives optimization of injection-moulding process for dashboard using soft computing and particle swarm optimization
Source: Sci Rep. 2024 Oct 10;14:23767. doi: 10.1038/s41598-024-62618-7 (PMC11467200; doi:10.1038/s41598-024-62618-7)
Supplement: Supplementary file 1 — Supplementary Information. [file 41598_2024_62618_MOESM1_ESM.docx]

The Extracted Data Set Using FEM:

| Index | T_1_ | T_2_ | t_1_ | t_2_ | Shrinkage (%) | Warpage (mm) | Sink mark (mm) |
| --- | --- | --- | --- | --- | --- | --- | --- |
| 1 | 200 | 25 | 10 | 60 | 5.15 | 5.97 | 0.0172 |
| 2 | 230 | 25 | 10 | 60 | 7.1 | 6.98 | 0.0207 |
| 3 | 280 | 25 | 10 | 60 | 9.81 | 9.05 | 0.0259 |
| 4 | 200 | 50 | 10 | 60 | 5.57 | 5.3 | 0.0176 |
| 5 | 230 | 50 | 10 | 60 | 7.38 | 6.25 | 0.0205 |
| 6 | 280 | 50 | 10 | 60 | 9.99 | 8.27 | 0.0253 |
| 7 | 200 | 80 | 10 | 60 | 5.79 | 4.64 | 0.016 |
| 8 | 230 | 80 | 10 | 60 | 7.67 | 5.57 | 0.0185 |
| 9 | 280 | 80 | 10 | 60 | 10.21 | 7.12 | 0.0224 |
| 10 | 200 | 25 | 20 | 60 | 4.99 | 6.95 | 0.0172 |
| 11 | 230 | 25 | 20 | 60 | 7 | 8.08 | 0.0207 |
| 12 | 280 | 25 | 20 | 60 | 9.67 | 10.37 | 0.0259 |
| 13 | 200 | 50 | 20 | 60 | 5.47 | 6.11 | 0.0176 |
| 14 | 230 | 50 | 20 | 60 | 7.28 | 7.21 | 0.0205 |
| 15 | 280 | 50 | 20 | 60 | 9.87 | 9.44 | 0.0253 |
| 16 | 200 | 80 | 20 | 60 | 5.7 | 4.64 | 0.016 |
| 17 | 230 | 80 | 20 | 60 | 7.57 | 5.88 | 0.0185 |
| 18 | 280 | 80 | 20 | 60 | 10.12 | 8.08 | 0.0224 |
| 19 | 200 | 25 | 30 | 60 | 4.86 | 7.39 | 0.0172 |
| 20 | 230 | 25 | 30 | 60 | 6.9 | 8.49 | 0.0207 |
| 21 | 280 | 25 | 30 | 60 | 9.6 | 10.97 | 0.0259 |
| 22 | 200 | 50 | 30 | 60 | 5.39 | 6.42 | 0.0176 |
| 23 | 230 | 50 | 30 | 60 | 7.2 | 7.56 | 0.0205 |
| 24 | 280 | 50 | 30 | 60 | 9.79 | 9.85 | 0.0253 |
| 25 | 200 | 80 | 30 | 60 | 5.63 | 4.82 | 0.016 |
| 26 | 230 | 80 | 30 | 60 | 7.51 | 6.15 | 0.0185 |
| 27 | 280 | 80 | 30 | 60 | 10.04 | 8.41 | 0.0224 |
| 28 | 200 | 25 | 10 | 80 | 5.14 | 6.11 | 0.0172 |
| 29 | 230 | 25 | 10 | 80 | 7.1 | 7.1 | 0.0207 |
| 30 | 280 | 25 | 10 | 80 | 9.81 | 9.2 | 0.0259 |
| 31 | 200 | 50 | 10 | 80 | 5.57 | 5.4 | 0.0176 |
| 32 | 230 | 50 | 10 | 80 | 7.38 | 6.37 | 0.0205 |
| 33 | 280 | 50 | 10 | 80 | 9.99 | 8.41 | 0.0253 |
| 34 | 200 | 80 | 10 | 80 | 5.79 | 4.66 | 0.016 |
| 35 | 230 | 80 | 10 | 80 | 7.67 | 5.59 | 0.0185 |
| 36 | 280 | 80 | 10 | 80 | 10.21 | 7.23 | 0.0224 |
| 37 | 200 | 25 | 20 | 80 | 4.98 | 7.08 | 0.0172 |
| 38 | 230 | 25 | 20 | 80 | 7 | 8.19 | 0.0207 |
| 39 | 280 | 25 | 20 | 80 | 9.68 | 10.48 | 0.0259 |
| 40 | 200 | 50 | 20 | 80 | 5.47 | 6.19 | 0.0176 |
| 41 | 230 | 50 | 20 | 80 | 7.28 | 7.29 | 0.0205 |
| 42 | 280 | 50 | 20 | 80 | 9.87 | 9.53 | 0.0253 |
| 43 | 200 | 80 | 20 | 80 | 5.7 | 4.65 | 0.016 |
| 44 | 230 | 80 | 20 | 80 | 7.57 | 5.96 | 0.0185 |
| 45 | 280 | 80 | 20 | 80 | 10.12 | 8.16 | 0.0224 |
| 46 | 200 | 25 | 30 | 80 | 4.85 | 7.45 | 0.0172 |
| 47 | 230 | 25 | 30 | 80 | 6.9 | 8.59 | 0.0207 |
| 48 | 280 | 25 | 30 | 80 | 9.58 | 10.92 | 0.0259 |
| 49 | 200 | 50 | 30 | 80 | 5.39 | 6.49 | 0.0176 |
| 50 | 230 | 50 | 30 | 80 | 7.2 | 7.62 | 0.0205 |
| 51 | 280 | 50 | 30 | 80 | 9.79 | 9.91 | 0.0253 |
| 52 | 200 | 80 | 30 | 80 | 5.63 | 4.86 | 0.016 |
| 53 | 230 | 80 | 30 | 80 | 7.51 | 6.21 | 0.0185 |
| 54 | 280 | 80 | 30 | 80 | 10.04 | 8.47 | 0.0224 |
| 55 | 200 | 25 | 10 | 100 | 5.13 | 6.2 | 0.0172 |
| 56 | 230 | 25 | 10 | 100 | 7.1 | 7.18 | 0.0207 |
| 57 | 280 | 25 | 10 | 100 | 9.81 | 9.28 | 0.0259 |
| 58 | 200 | 50 | 10 | 100 | 5.57 | 5.47 | 0.0176 |
| 59 | 230 | 50 | 10 | 100 | 7.38 | 6.43 | 0.0205 |
| 60 | 280 | 50 | 10 | 100 | 9.99 | 8.47 | 0.0253 |
| 61 | 200 | 80 | 10 | 100 | 5.79 | 4.66 | 0.016 |
| 62 | 230 | 80 | 10 | 100 | 7.67 | 5.6 | 0.0185 |
| 63 | 280 | 80 | 10 | 100 | 7.38 | 6.37 | 0.0205 |
| 64 | 200 | 25 | 20 | 100 | 4.98 | 7.16 | 0.0172 |
| 65 | 230 | 25 | 20 | 100 | 7.38 | 6.37 | 0.0205 |
| 66 | 280 | 25 | 20 | 100 | 9.68 | 10.53 | 0.0259 |
| 67 | 200 | 50 | 20 | 100 | 5.47 | 6.25 | 0.0176 |
| 68 | 230 | 50 | 20 | 100 | 7.28 | 7.34 | 0.0205 |
| 69 | 280 | 50 | 20 | 100 | 9.87 | 9.56 | 0.0253 |
| 70 | 200 | 80 | 20 | 100 | 5.7 | 4.67 | 0.016 |
| 71 | 230 | 80 | 20 | 100 | 5.57 | 5.99 | 0.0185 |
| 72 | 280 | 80 | 20 | 100 | 10.12 | 8.19 | 0.0224 |
| 73 | 200 | 25 | 30 | 100 | 4.85 | 7.53 | 0.0172 |
| 74 | 230 | 25 | 30 | 100 | 6.91 | 8.77 | 0.0207 |
| 75 | 280 | 25 | 30 | 100 | 9.58 | 10.97 | 0.0259 |
| 76 | 200 | 50 | 30 | 100 | 5.39 | 6.54 | 0.0176 |
| 77 | 230 | 50 | 30 | 100 | 7.206 | 7.66 | 0.0205 |
| 78 | 280 | 50 | 30 | 100 | 9.79 | 9.92 | 0.0253 |
| 79 | 200 | 80 | 30 | 100 | 5.63 | 4.88 | 0.016 |
| 80 | 230 | 80 | 30 | 100 | 7.51 | 6.23 | 0.0185 |
| 81 | 280 | 80 | 30 | 100 | 10.04 | 8.48 | 0.0224 |
| 82 | 215 | 25 | 10 | 60 | 6.207 | 6.25 | 0.019 |
| 83 | 215 | 25 | 10 | 80 | 6.17 | 6.62 | 0.019 |
| 84 | 215 | 25 | 10 | 100 | 6.2 | 6.74 | 0.019 |
| 85 | 215 | 25 | 20 | 60 | 6.02 | 7.52 | 0.019 |
| 86 | 215 | 25 | 20 | 80 | 6.03 | 7.66 | 0.019 |
| 87 | 215 | 25 | 20 | 100 | 6.03 | 7.74 | 0.019 |
| 88 | 215 | 25 | 30 | 60 | 5.93 | 7.93 | 0.019 |
| 89 | 215 | 25 | 30 | 80 | 5.93 | 8.04 | 0.019 |
| 90 | 215 | 25 | 30 | 100 | 5.93 | 8.11 | 0.019 |
| 91 | 215 | 50 | 10 | 60 | 6.83 | 5.12 | 0.0174 |
| 92 | 215 | 50 | 10 | 80 | 6.47 | 5.82 | 0.0189 |
| 93 | 215 | 50 | 10 | 100 | 6.47 | 5.88 | 0.0189 |
| 94 | 215 | 50 | 20 | 60 | 6.81 | 6.66 | 0.0193 |
| 95 | 215 | 50 | 20 | 80 | 6.81 | 6.76 | 0.0193 |
| 96 | 215 | 50 | 20 | 100 | 6.36 | 6.74 | 0.0189 |
| 97 | 215 | 50 | 30 | 60 | 6.28 | 6.93 | 0.0189 |
| 98 | 215 | 50 | 30 | 80 | 6.28 | 6.99 | 0.0189 |
| 99 | 215 | 50 | 30 | 100 | 6.28 | 7.04 | 0.0189 |
| 100 | 215 | 80 | 10 | 60 | 6.83 | 5.12 | 0.0174 |
| 101 | 215 | 80 | 10 | 80 | 6.83 | 5.14 | 0.0174 |
| 102 | 215 | 80 | 10 | 100 | 6.83 | 5.14 | 0.0174 |
| 103 | 215 | 80 | 20 | 60 | 6.72 | 5.24 | 0.0174 |
| 104 | 215 | 80 | 20 | 80 | 6.72 | 5.3 | 0.0174 |
| 105 | 215 | 80 | 20 | 100 | 6.72 | 5.34 | 0.0174 |
| 106 | 215 | 80 | 30 | 60 | 6.66 | 5.34 | 0.0174 |
| 107 | 215 | 80 | 30 | 80 | 6.66 | 5.54 | 0.0174 |
| 108 | 215 | 80 | 30 | 100 | 6.66 | 5.56 | 0.0174 |
| 109 | 215 | 65 | 10 | 60 | 6.65 | 5.26 | 0.0186 |
| 110 | 215 | 65 | 10 | 80 | 6.65 | 5.26 | 0.0186 |
| 111 | 215 | 65 | 10 | 100 | 6.65 | 5.31 | 0.0186 |
| 112 | 215 | 65 | 20 | 60 | 6.54 | 5.95 | 0.0186 |
| 113 | 215 | 65 | 20 | 80 | 6.54 | 6.02 | 0.0186 |
| 114 | 215 | 65 | 20 | 100 | 6.54 | 5.06 | 0.0186 |
| 115 | 215 | 65 | 30 | 60 | 6.47 | 6.24 | 0.0186 |
| 116 | 215 | 65 | 30 | 80 | 6.47 | 6.29 | 0.0186 |
| 117 | 215 | 65 | 30 | 100 | 6.47 | 6.29 | 0.0186 |
| 118 | 255 | 25 | 10 | 60 | 8.59 | 8.23 | 0.0235 |
| 119 | 255 | 25 | 10 | 80 | 8.59 | 8.08 | 0.0235 |
| 120 | 255 | 25 | 10 | 100 | 8.59 | 8.08 | 0.0235 |
| 121 | 255 | 25 | 20 | 60 | 8.47 | 8.08 | 0.0235 |
| 122 | 255 | 25 | 20 | 80 | 4.92 | 8.47 | 0.0235 |
| 123 | 255 | 25 | 20 | 100 | 8.47 | 9.33 | 0.0235 |
| 124 | 255 | 25 | 30 | 60 | 8.37 | 9.33 | 0.0235 |
| 125 | 255 | 25 | 30 | 80 | 8.37 | 9.7 | 0.0235 |
| 126 | 255 | 25 | 30 | 100 | 8.37 | 9.75 | 0.0235 |
| 127 | 255 | 50 | 10 | 60 | 8.74 | 7.24 | 0.023 |
| 128 | 255 | 50 | 10 | 80 | 8.74 | 7.37 | 0.023 |
| 129 | 255 | 50 | 10 | 100 | 8.74 | 7.37 | 0.023 |
| 130 | 255 | 50 | 20 | 60 | 8.63 | 7.37 | 0.023 |
| 131 | 255 | 50 | 20 | 80 | 8.63 | 7.37 | 0.023 |
| 132 | 255 | 50 | 20 | 100 | 8.63 | 8.42 | 0.023 |
| 133 | 255 | 50 | 30 | 60 | 8.55 | 8.68 | 0.023 |
| 134 | 255 | 50 | 30 | 80 | 8.74 | 8.55 | 0.023 |
| 135 | 255 | 50 | 30 | 100 | 8.55 | 8.77 | 0.023 |
| 136 | 255 | 80 | 10 | 60 | 8.98 | 6.31 | 0.0205 |
| 137 | 255 | 80 | 10 | 80 | 8.98 | 6.33 | 0.0205 |
| 138 | 255 | 80 | 10 | 100 | 8.98 | 6.33 | 0.0205 |
| 139 | 255 | 80 | 20 | 60 | 8.88 | 6.95 | 0.0205 |
| 140 | 255 | 80 | 20 | 80 | 8.88 | 7.02 | 0.0205 |
| 141 | 255 | 80 | 20 | 100 | 8.88 | 7.05 | 0.0205 |
| 142 | 255 | 80 | 30 | 60 | 8.81 | 7.25 | 0.0205 |
| 143 | 255 | 80 | 30 | 80 | 8.81 | 7.3 | 0.0205 |
| 144 | 255 | 80 | 30 | 100 | 8.81 | 7.32 | 0.0205 |
| 145 | 255 | 65 | 10 | 60 | 8.88 | 6.66 | 0.0222 |
| 146 | 255 | 65 | 10 | 80 | 8.88 | 6.78 | 0.0222 |
| 147 | 255 | 65 | 10 | 100 | 8.88 | 6.83 | 0.0222 |
| 148 | 255 | 65 | 20 | 60 | 8.77 | 7.62 | 0.0222 |
| 149 | 255 | 65 | 20 | 80 | 8.77 | 7.7 | 0.0222 |
| 150 | 255 | 65 | 20 | 100 | 8.77 | 7.73 | 0.0222 |
| 151 | 255 | 65 | 30 | 60 | 8.69 | 7.73 | 0.0222 |
| 152 | 255 | 65 | 30 | 80 | 8.69 | 8.02 | 0.0222 |
| 153 | 255 | 65 | 30 | 100 | 8.69 | 8.03 | 0.0222 |
| 154 | 200 | 65 | 10 | 60 | 5.69 | 4.78 | 0.017 |
| 155 | 200 | 65 | 10 | 80 | 5.69 | 4.8 | 0.017 |
| 156 | 200 | 65 | 10 | 100 | 5.69 | 4.79 | 0.017 |
| 157 | 200 | 65 | 20 | 60 | 5.58 | 5.31 | 0.017 |
| 158 | 200 | 65 | 20 | 80 | 5.58 | 5.37 | 0.017 |
| 159 | 200 | 65 | 20 | 100 | 5.58 | 5.41 | 0.017 |
| 160 | 200 | 65 | 30 | 60 | 5.48 | 5.59 | 0.017 |
| 161 | 200 | 65 | 30 | 80 | 5.48 | 5.63 | 0.017 |
| 162 | 200 | 65 | 30 | 100 | 5.48 | 5.66 | 0.017 |
| 163 | 230 | 65 | 10 | 60 | 7.55 | 5.7 | 0.0199 |
| 164 | 230 | 65 | 10 | 80 | 7.55 | 5.81 | 0.0199 |
| 165 | 230 | 65 | 10 | 100 | 7.55 | 5.87 | 0.0199 |
| 166 | 230 | 65 | 20 | 60 | 7.45 | 6.55 | 0.0199 |
| 167 | 230 | 65 | 20 | 80 | 7.45 | 6.63 | 0.0199 |
| 168 | 230 | 65 | 20 | 100 | 7.45 | 6.67 | 0.0199 |
| 169 | 230 | 65 | 30 | 60 | 7.37 | 6.88 | 0.0199 |
| 170 | 230 | 65 | 30 | 80 | 7.37 | 6.92 | 0.0199 |
| 171 | 230 | 65 | 30 | 100 | 7.37 | 6.95 | 0.0199 |
| 172 | 250 | 65 | 10 | 60 | 8.61 | 6.47 | 0.0218 |
| 173 | 250 | 65 | 10 | 80 | 8.63 | 6.59 | 0.0218 |
| 174 | 250 | 65 | 10 | 100 | 8.63 | 6.64 | 0.0218 |
| 175 | 250 | 65 | 20 | 60 | 8.53 | 7.41 | 0.0218 |
| 176 | 250 | 65 | 20 | 80 | 8.53 | 7.49 | 0.0218 |
| 177 | 250 | 65 | 20 | 100 | 8.53 | 7.52 | 0.0218 |
| 178 | 250 | 65 | 30 | 60 | 8.45 | 7.74 | 0.0218 |
| 179 | 250 | 65 | 30 | 80 | 8.45 | 7.8 | 0.0218 |
| 180 | 250 | 65 | 30 | 100 | 8.45 | 7.82 | 0.0218 |
